# Squaramides and Ureas: A Flexible Approach to Polymerase‐Compatible Nucleic Acid Assembly

**DOI:** 10.1002/anie.202000209

**Published:** 2020-05-07

**Authors:** Arun Shivalingam, Lapatrada Taemaitree, Afaf H. El‐Sagheer, Tom Brown

**Affiliations:** ^1^ Department of Chemistry University of Oxford Chemistry Research Laboratory 12 Mansfield Road Oxford OX1 3TA UK; ^2^ Department of Science and Mathematics Suez University Faculty of Petroleum and Mining Engineering Suez 43721 Egypt

**Keywords:** ligation, nucleic acids, polymerase chain reaction, RNA detection, squaramide

## Abstract

Joining oligonucleotides together (ligation) is a powerful means of retrieving information from the nanoscale. To recover this information, the linkages created must be compatible with polymerases. However, enzymatic ligation is restrictive and current chemical ligation methods lack flexibility. Herein, a versatile ligation platform based on the formation of urea and squaramide artificial backbones from minimally modified 3′‐ and 5′‐amino oligonucleotides is described. One‐pot ligation gives a urea linkage with excellent read‐through speed, or a squaramide linkage that is read‐through under selective conditions. The squaramide linkage can be broken and reformed on demand, while stable pre‐activated precursor oligonucleotides expand the scope of the ligation reaction to reagent‐free, mild conditions. The utility of our system is demonstrated by replacing the enzymatically biased RNA‐to‐DNA reverse transcription step of RT‐qPCR with a rapid nucleic‐acid‐template‐dependent DNA chemical ligation system, that allows direct RNA detection.

## Graphical abstract

**3′‐ and 5′‐amino oligonucleotides** are chemically ligated through the formation of urea and squaramide artificial backbones. The squaramide linkage can be formed in mild reagent‐free buffered conditions, read‐through accurately by specific polymerases, and even cleaved and reformed on demand. To demonstrate its utility, the RNA‐to‐DNA reverse transcription step of RT‐qPCR is replaced with squaramide chemical ligation for direct RNA detection.

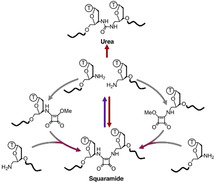

## Introduction

**Figure 1:**
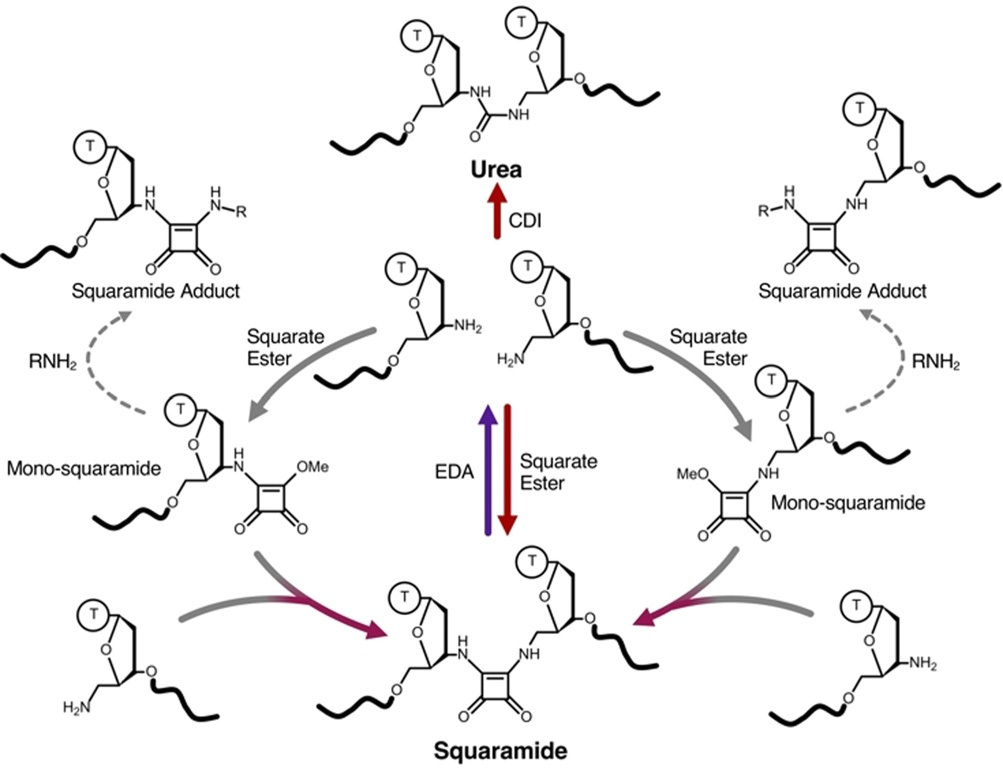
Overview of an oligonucleotide ligation platform that generates read‐through‐competent artificial squaramide and urea backbones. The squaramide linkage can be formed and cleaved under selective conditions. The chemical ligation of the 3′‐ and 5′‐amino starting oligonucleotides into a urea linkage is also feasible. CDI=1,1′‐carbonyldiimidazole. EDA=ethylenediamine. Squarate ester=3,4‐dimethoxy‐3‐cyclobutene‐1,2‐dione.

Oligonucleotides provide the ideal means of storing information at the molecular level. From barcoding compounds in high‐throughput drug discovery,^1^, ^2^, ^3^, ^4^ directing the motion of molecular machines,^5^, ^6^ and even “bio‐hacking” computer software,^7^ writing instructions or messages using oligonucleotides has become established practice. However, a significant limitation is our dependence on ligase enzymes to join oligonucleotide strands together; these enzymes require highly specific reaction conditions and substrates. Consequently purely chemical nucleic acid ligation methods have been explored;^8^, ^9^ amide,^10^ CuAAC,^11^, ^12^, ^13^ phosphoramidate (PA),^14^, ^15^ phosphorothioate (PS),^16^, ^17^, ^18^ and thiol–thiol^19^, ^20^ coupling reactions generate artificial backbones that are recognised and read‐through by DNA polymerases, thereby enabling information retrieval. Yet all face potential drawbacks, including copper dependence (CuAAC), precursor handling (disulfide, PA, PS), slow ligation rates (PS), and poor read‐through fidelity (disulfide). This leaves scope for artificial backbones that synergise all their advantageous properties, including excellent fidelity, reversibility (disulfide), and pre‐activation (PA, PS). To this end, we describe a versatile ligation platform based on oligonucleotides containing commercially available 3′‐ and 5′‐amino‐modified resins and phosphoramidites, respectively (Figure 1). Stable (urea) or chemically reversible (squaramide) artificial linkages are generated, which can be read‐through by polymerase enzymes (replicated) under selective conditions. Through the formation of stable pre‐activated precursor oligonucleotides, a simple process, more controlled ligation can occur in reagent‐free, mild buffered conditions. The ease and utility of our system is demonstrated by replacing the reverse transcription (RT) step in RT‐qPCR with RNA‐templated squaramide ligation of DNA; a favourable approach that removes known enzymatic‐protocol‐dependent biases,^21^, ^22^, ^23^ provides the inherent target specificity required for hybridisation of two adjacent oligonucleotides, and minimises genomic DNA contamination effects through the use of “tailed” ligation oligonucleotides for subsequent PCR amplification.

Our inspiration came from the excellent read‐through fidelity of an artificial amide backbone,^10^ and the various nucleic acid backbone analogues that have been reported for RNA therapeutic applications. Structurally related carbamate,^24^, ^25^, ^26^ thiourea,^27^ urea,^26^ and squaramides^28^ have been introduced into oligonucleotides by iterative coupling or as phosphoramidite dinucleotides, but neither their formation by templated ligation nor their read‐through fidelity have been explored. Interestingly, all of these backbones could be derived from unmodified or minimally modified oligonucleotides where the terminal 5′‐ or 3′‐hydroxy groups are replaced by amino groups. Furthermore, the reagents (for example, 1,1′‐carbonyldiimidazole (CDI) and squarate ester) used to generate these linkages become progressively less reactive upon each nucleophilic addition‐elimination step,^29^ potentially allowing oligonucleotides to be pre‐activated prior to ligation with another strand.

## Results and Discussion

**Figure 2:**
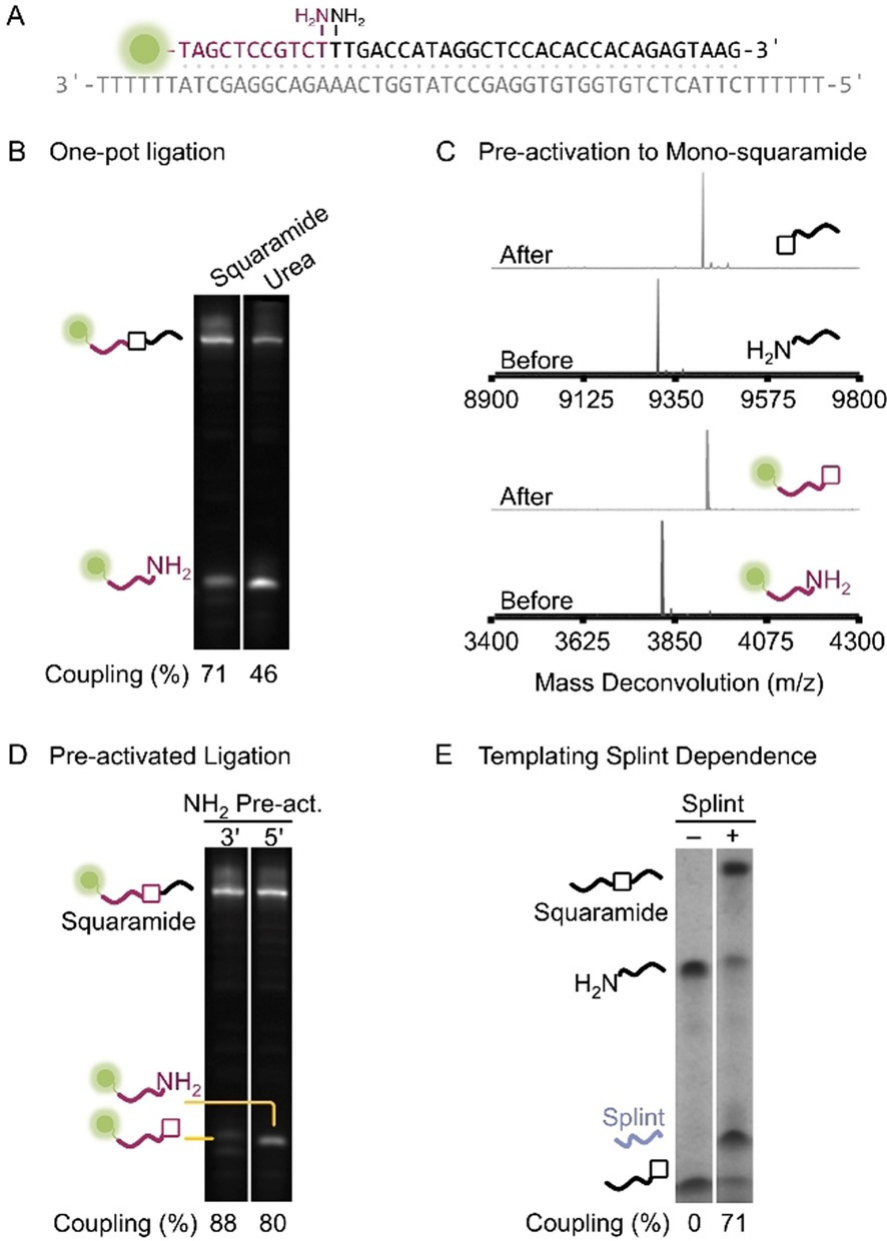
Ligation of 3′‐ and 5′‐amino oligonucleotides to generate squaramide and urea linkages. A) Oligonucleotides used and their hybridisation for (B) to (D). B) One‐pot ligation, where all necessary oligonucleotides including splint are mixed together and squarate ester or CDI are added to generate squaramide and urea artificial backbones, respectively. The 3′‐amino oligonucleotide was labelled with a 5′‐FAM, which was used for denaturing PAGE visualisation and quantification of coupling by ImageJ analysis. Note coupling=f_product_/f_total_×100, where f=fraction. C) High‐concentration (100 μm) pre‐activation of 3′‐ and 5′‐amino oligonucleotides using squarate ester to generate mono‐squaramides. Mass spectrometry demonstrates complete activation and no dimerisation. *m*/*z* (5′‐FAM‐3′‐amino F1)=3819 (expected), 3819 (found); *m*/*z* (5′‐FAM‐3′‐mono‐squaramide F1)=3929 (expected), 3929 (found); *m*/*z* (5′‐amino Am3)=9305 (expected), 9305 (found); *m*/*z* (5′‐mono‐squaramide Am3)=9415 (expected), 9415 (found). D) Ligation when pre‐activated mono‐squaramide oligonucleotides are mixed with the appropriate amino oligonucleotide and splint. The gel was visualised and quantified as described for (B). E) The importance of a templating strand (splint) to bring the reactants together for squaramide chemical ligation when a pre‐activated mono‐squaramide oligonucleotide is used. The gel is visualised by UV shadowing. Note coupling=(1−f_unreacted SM_/f_total SM_)×100, where f=fraction and SM=starting material. The oligonucleotides used for (B) to (D) are listed in Table S1 and for (E) in Table S4 in the Supporting Information. A full summary of squaramide ligation efficiency can be found in Table S10 in the Supporting Information.

To investigate the feasibility of our ligation strategy, oligonucleotides bearing a single terminal 3′‐amino dT and 5′‐amino dT were prepared using conventional solid‐phase oligonucleotide synthetic methods and commercial resins and monomers, which provided the modified oligonucleotides in good yields and purity.^30^, ^31^ Next, “one‐pot” ligation of appropriate pairs of oligonucleotides was performed using a complementary templating DNA strand (splint) at pH 8.5. Under these conditions, the amine–ammonium ion equilibrium is shifted favourably to the amino form, whilst the templating strand significantly enhances the second nucleophilic addition‐elimination step by reducing its concentration dependence. Unfortunately, no evidence for the formation of carbamate or thiourea linkages was observed using CDI or its thio‐derivative (Supporting Information, Figure S1). On the other hand, squaramide‐ and urea‐linked oligonucleotide ligation proceeded well using squarate ester and CDI, respectively (71 % and 46 %, respectively, Figure 2 B). The difference in CDI reactivity for the carbamate and urea is likely due to the lower nucleophilicity of the hydroxy group required for carbamate formation (compare with the amino groups for the urea linkage). For the squaramide, ligation efficiency was dependent on pH (best to worst: 8.5>7.5>unbuffered, Supporting Information, Figure S2) and squarate methyl ester concentration (optimal=5 mm). For the urea, CDI was used as a solid in large excess, making optimisation of the urea ligation conditions challenging as the imidazole by‐product naturally buffers the system to neutral pH.

Mass spectrometry of the crude squaramide formation reaction mixture showed that residual amino oligonucleotides were quantitatively converted to mono‐squaramide monoesters. Therefore, pre‐activation of either the 3′‐ or 5′‐amino oligonucleotides was investigated before templated ligation to the second strand. Using identical conditions to the “one‐pot” ligation reaction and simple desalting with no further purification, pre‐activation was straightforward, no amino oligonucleotide dimerisation was observed and near‐quantitative activation was achieved even at very high (100 μm) oligonucleotide concentrations (Figure 2 C). When added to the appropriate amino oligonucleotides, good conversion to the squaramide was observed within 15 min if a templating strand was present (Figure 2 E). The 5′‐amino pre‐activated oligonucleotides gave slightly lower ligation efficiency (80 % vs. 88 % for the pre‐activated 3′‐amino oligonucleotide, Figure 2 D, a full summary of squaramide ligation efficiency can be found in Table S10 in the Supporting Information), most likely due to the 3′‐amino group being a more sterically hindered nucleophile.

Pleasingly these pre‐activated oligonucleotides were stable to hindered amines such as tris(hydroxymethyl)aminomethane (Tris) that is used in buffers, but could be inactivated in the presence of primary amines, such as ethanolamine (Supporting Information, Figure S3). For assembly of larger DNA constructs, it would be necessary to have oligonucleotides with both terminal 3′‐ and 5′‐amino groups. Pre‐activation in this case should avoid cyclic oligonucleotide formation but maintain activation of both terminal amino groups, which was indeed observed (Supporting Information, Figure S4). Surprisingly RNA oligonucleotides required higher squarate ester concentrations for complete pre‐activation (approximately 10 mm squarate ester, Supporting Information, Figure S4). Assembly of large nucleic acids would require annealing of multiple oligonucleotides, thus requiring the mono‐squaramide monoesters to have reasonable temperature stability. When heated for 5 min at 95 °C in standard Taq polymerase buffer (10 mm Tris‐HCl, 50 mm KCl, and 1.5 mm MgCl_2_, pH 8.3), 9 % and 18 % hydrolysis of the ester was observed for 5′‐ and 3′‐activated oligonucleotides, respectively (Supporting Information, Figure S5). However, decreasing the temperature (40 min, 55 °C, Supporting Information, Figure S5) reduces the hydrolysis rates to negligible levels.

Having established a robust ligation platform, the next focus was information retrieval. qPCR was performed using Phusion (exo+, exo=exonuclease activity) and Taq (exo‐) polymerases, the premise being that read‐through of the modified backbone to generate the unmodified complementary strand is rate‐determining on the amount of PCR product generated. Hence detection of PCR product at lower cycles is indicative of higher read‐through efficiency.

**Figure 3:**
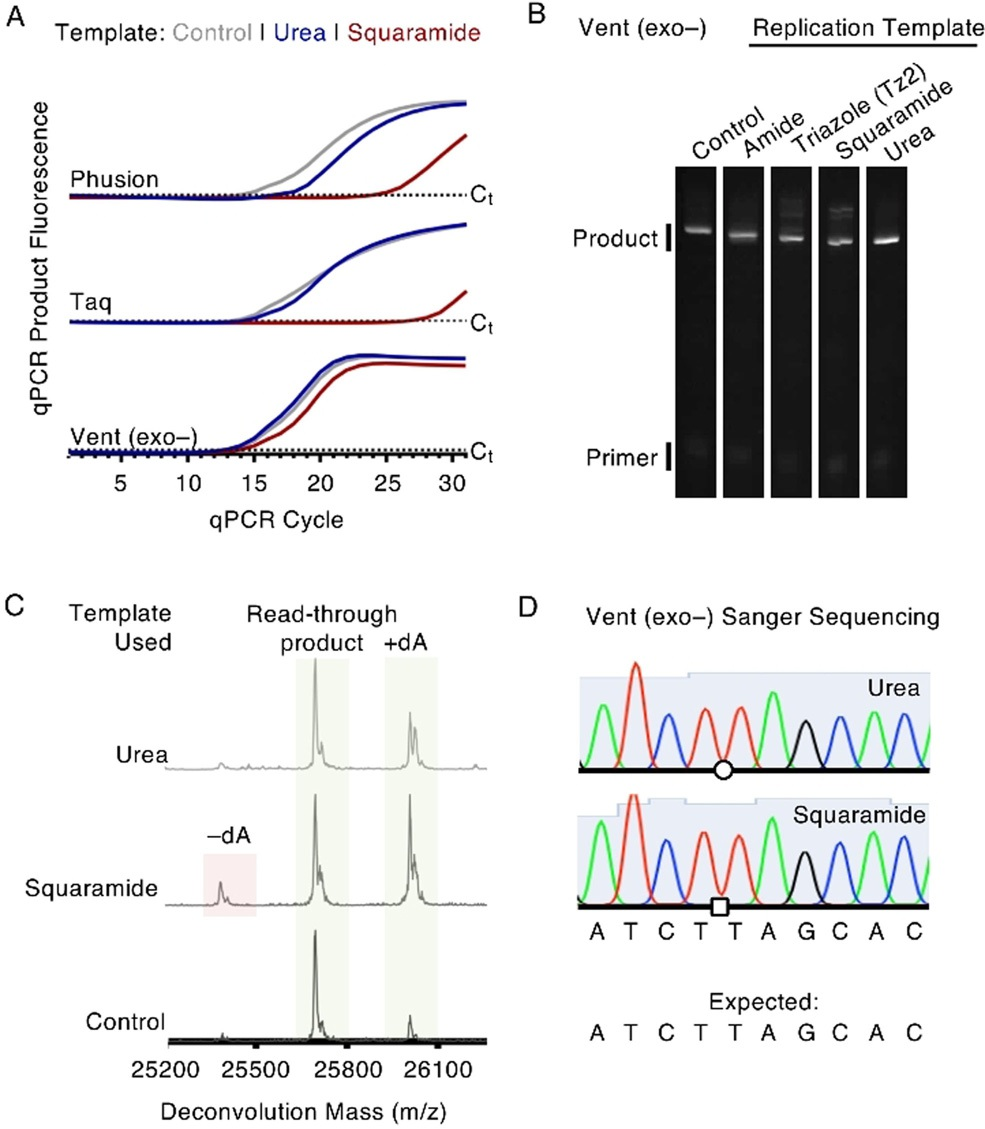
PCR and linear copying of artificial backbone‐containing oligonucleotides. A) qPCR curves for phosphodiester (control), urea, and squaramide backbones (colour‐coded) using hot‐start Taq, hot‐start flex Phusion or Vent (exo‐) polymerase. Urea backbones are tolerated well by all tested polymerases, while the squaramide backbone is selectively read‐through using Vent (exo‐) polymerase. B) Linear copying of templates containing artificial backbones using Vent (exo‐) polymerase for 1 h at 60 °C by denaturing PAGE. Previously reported triazole and amide backbones are shown for reference. C) Mass spectrometry analysis of the products from read‐though of squaramide‐ and urea‐containing oligonucleotides by Vent (exo‐) polymerase (2 h at 60 °C). *m*/*z* (read‐through product)=25696 (expected), 25696 (found). Note that due to a lack of proof‐reading activity, the polymerase can add an untemplated dA base (see phosphodiester control). Shoulder peaks are the result of salt adducts from the reaction buffer. D) Representative Sanger sequencing results for Vent (exo‐) PCR amplicons cloned into a vector. Urea and squaramide linkages are represented by circle and square symbols, respectively. Full alignments can be found in Figure S8 in the Supporting Information. The oligonucleotides used are listed in Tables S4–S7 in the Supporting Information.

Both polymerases tolerated the urea linkage well, with PCR product fluorescence reaching a detectable level after a number of cycles (cycle threshold, C_t_) comparable to that of the canonical phosphodiester linkage (Figure 3 A). Conversely, these polymerases were less tolerant of the squaramide linkage (Figure 3 A, ΔC_t_ control‐squaramide≈10 (Phusion) and ≈14 (Taq) cycles, which is equivalent to approximately 1000 and 10 000‐fold less PCR product, respectively assuming 100 % PCR efficiency). Optimisation of the PCR buffer to remove monovalent cations improved the situation but the amount of product was still significantly lower than the control (ΔC_t_ control‐squaramide≈10 (Taq), Supporting Information, Figure S6). Changing the polymerase enzyme, however, was the most effective solution. Remarkably, Vent (exo‐) gave comparable C_t_ values for control and squaramide backbones with no alteration to the buffer supplied by the manufacturer (Figure 3 A). To corroborate these results, linear copying of the modified templates and analysis by gel‐electrophoresis was performed using Vent (exo‐) polymerase, with DNA templates containing previously reported^10^ amide and triazole backbones providing points of reference (Figure 3 B). Read‐through of the amide and urea backbones was comparable, which is consistent with their similar structural demands. Squaramide and triazole backbone read‐through was also comparable for Vent (exo‐) polymerase, and demonstrates the importance of the polymerase; the triazole linkage has been extensively tested and shown to function with a range of polymerases (for example, Taq and Phusion),^10^ whereas the squaramide linkage is selective, with read‐through only being efficient for Vent (exo‐) polymerase.

Linear extension also allowed mass spectrometry characterisation of the read‐through products (Figure 3 C). For the unmodified control, the expected mass of the full‐length read‐through product was observed in addition to a peak for the product plus an additional dA nucleotide. This is consistent with the known tendency of polymerases lacking exonuclease (proof‐reading) activity to add an untemplated terminal dA nucleotide. For the squaramide template, the same products as the control were observed in addition to a product missing one dA nucleotide relative to the full‐length product, the exact location of which was probed by Sanger sequencing (see below). For the urea linkage, only correct full‐length and “full‐length plus dA” products were observed. Interestingly, the choice of polymerase is again important; in contrast to accurate read‐through with Vent (exo‐) polymerase, Klenow (exo+) polymerase generates an equal mixture of products lacking one or two dA nucleotides for the urea backbone, with negligible full‐length product (Supporting Information, Figure S7).

To verify the exact location of the dA insertions and deletions, PCR amplicons were generated using Vent (exo‐) polymerase and synthetic squaramide‐ or urea‐containing templates, cloned into a vector, and transformed into *Escherichia coli*. Several colonies were randomly picked (*n*=10 and 8 for squaramide and urea, respectively) and the recovered vectors sent for Sanger sequencing (Figure 3 D, full alignments in Figure S8 in the Supporting Information). All colonies, bar one, displayed correct base specificity adjacent to the artificial linkage, confirming the extra dA nucleotides are the result of the polymerase's tendency to add an untemplated terminal dA nucleotide. The one exception was a dT deletion directly adjacent to the site of the urea linkage, a rare occurrence (1/8).

The urea linkage also displayed four other single base mismatches but these were non‐conserved and occurred greater than 10 bases away from the modification site. For the squaramide, only one C‐to‐T mismatch mutation was observed 7 bases away from the modification, suggesting the dA deletion seen by mass spectrometry could be a terminal 3′ base deletion.

**Figure 4:**
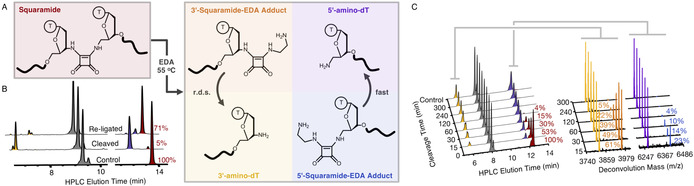
Cleavage and re‐ligation of the squaramide backbone. Note that oligonucleotides are colour‐coded in all graphs based on the text and box colours in the reaction Scheme in (A). A) The various products generated upon treatment of squaramide‐containing oligonucleotides with ethylenediamine. Conversion of the 3′‐squaramide‐ethylenediamine adduct to the free 3′‐amine is the rate‐determining step (r.d.s.) to regenerate the 5′‐amino and 3′‐amino oligonucleotides for re‐ligation. B) An example of squaramide cleavage (1:3 v/v EDA to oligonucleotide in water, 3 h at 55 °C) and re‐ligation (squarate ester in 15 mm sodium borate buffer, pH 8.5 containing 0.2 m NaCl). Each reaction was analysed by UPLC‐MS with the UPLC traces shown here. Relative amounts of the squaramide‐containing oligonucleotide were determined by integration of the traces, which were all normalised to the splint peak intensity (grey). C) Time‐dependent cleavage of squaramide‐containing oligonucleotides using 1:3 v/v of ethylenediamine to oligonucleotide in water at 55 °C. Both UPLC traces and mass spectrometry deconvolution of these peaks are shown to highlight that the removal of the 3′‐squaramide‐EDA adduct is rate limiting in obtaining the free amino oligonucleotides. UPLC peaks were integrated to determine relative conversion of the squaramide‐adduct oligonucleotide to the free amino oligonucleotides. Note that all traces are normalised to the splint peak intensity (grey). For mass spectrometry, the ratio of deconvoluted masses is shown to estimate squaramide‐adduct amounts. This assumes ionisation of the free amino oligonucleotide and squaramide‐ethylenediamine adduct is comparable and dominated by the negative charge of the oligonucleotide. *m/z* (3′‐squaramide‐EDA adduct)=3957 (expected), 3957 (found); *m*/*z* (5′‐squaramide‐EDA adduct)=6405 (expected), 6406 (found). The oligonucleotides used for (B) are listed in Supplementary Table 2 and for (C) in Supplementary Table 1.

Squaramide‐containing oligonucleotides have been reported to be susceptible to hydrolysis during ammonia‐mediated nucleobase deprotection.^28^ This raises the possibility of excising the squaramide linkage and regenerating the oligonucleotide starting materials under appropriate conditions. To evaluate this, a range of nucleophiles (methylamine, ethanolamine, ethylenediamine, cysteamine, and dithiothreitol) were screened for their ability to break the squaramide linkage in 1 h at 55 °C (Supporting Information, Figure S9). Thiols were ineffective, however primary amines showed appreciable squaramide backbone cleavage. Ethylenediamine was the most effective reagent (Figure 4). This is likely due to the initial products formed upon cleavage; nucleophilic addition‐elimination of the amine on the squaramide‐ligated oligonucleotide generates one strand with a free amino group and another strand with the remaining squaramide‐ethylenediamine adduct (Figure 4 A). Intramolecular attack of the free amino group of ethylenediamine is likely to facilitate the second nucleophilic addition‐elimination reaction to release the squaramide from the oligonucleotide. The identity of the oligonucleotide adducts was confirmed by UPLC‐MS (Figure 4 C). Following the reaction over time indicated that the rate determining step to recover the starting material was removal of the squaramide‐ethylenediamine adduct from the 3′‐amino terminus of the oligonucleotide. Optimised cleavage conditions involved the addition of 50 % aqueous ethylenediamine to an equal volume of aqueous oligonucleotide sample. Heating for 3 h at 55 °C gave approximately 95 % cleavage of the linkage (Figure 4 B). Confirmation that the starting material had been recovered was demonstrated by desalting the sample by gel‐filtration, which also removes ethylenediamine, freeze‐drying, and re‐ligating the oligonucleotides using one‐pot ligation conditions (Figure 4 B). Pleasingly, a comparable coupling yield to that observed during the ligation optimisation (Figure 2 B) was observed.

**Figure 5:**
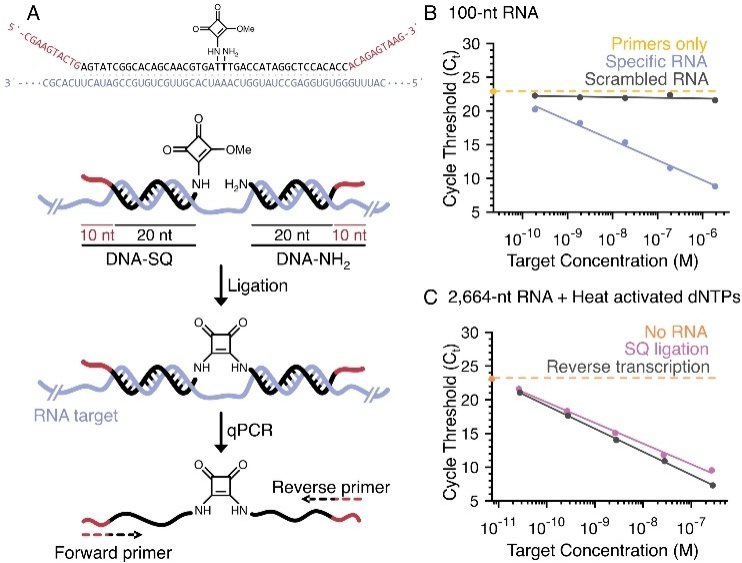
RNA detection through templated squaramide ligation and qPCR. A) The sequence of the oligonucleotides used for (B) and the overall design principle of the chemical‐ligation detection system. Two DNA oligonucleotides, one a pre‐activated 3′‐mono‐squaramide and the other a free 5′‐amino group, must hybridise adjacent to each other on the target RNA in order to initiate ligation. These DNA oligonucleotides contain 10‐base “tails” that distinguish the resulting template from genomic DNA. Primers that hybridise to the resultant template then amplify the sequence for quantitative PCR detection using the dye EvaGreen. The key benefits are direct validation of the target sequence before PCR (two oligonucleotides of approximately 20 bases must hybridise adjacent to each other for ligation and therefore RNA detection) and the lack of enzyme (polymerase or ligase)/ primer bias in RNA‐to‐DNA conversion. B) The squaramide ligation system is responsive only to the target RNA sequence and not the scrambled control. The limitation being primer‐only amplification. C) The system performs comparably to RT‐qPCR over the same dynamic range (0.3 μm to 30 pm, five‐orders of magnitude) for a 2664‐mer target RNA spiked into 100 ng total RNA isolated from MCF‐7 cells. Through the use of heat‐activated dNTPs, the limitation of detection is untemplated squaramide ligation (namely, no RNA template, see Figure S10 B in the Supporting Information for negligible primer‐only amplification). The sequences used for (B) and (C) are in Table S9 in the Supporting Information.

Finally, to demonstrate the utility of our enzyme‐free ligation platform, we focussed on RNA detection. RT‐qPCR is the current gold‐standard for RNA detection but the methodology has several limitations: 1) the priming strategy used for RNA‐to‐cDNA conversion (for example, random hexamers vs. poly(dT) primers) is known to influence the results obtained;^21^, ^22^, ^23^ 2) genomic DNA contamination remains an issue even with DNase‐treated RNA samples;^32^ 3) the identity of the PCR product needs to be validated during or post‐PCR (typically using an oligonucleotide that hybridises to the PCR product). We envisaged a ligation‐dependent qPCR design (Figure 5 A) that could address these points. Our design requires two DNA oligonucleotides to hybridise next to each other in buffered, reagent‐free conditions to form the template that will be used for qPCR. This validates the identity of the target RNA prior to PCR directly from the unmodified source sample. More importantly, no polymerase‐priming strategy is required as the RNA‐to‐DNA conversion relies solely on hybridisation; enzymatic bias in template read‐through and/or secondary structure interference are therefore minimised.^33^ Since the DNA oligonucleotides will be directly used for qPCR, the introduction of “tails” (sequences) that are not found naturally in the genome enables the design of primers that are highly specific to the DNA template. Similar in concept to Padlock probes,^34^, ^35^ the key differentiator is that squaramide chemical ligation enables efficient ligation irrespective of nucleic acid substrate, allowing RNA detection that is otherwise challenging with ligase enzymes.^36^

In designing our pair of RNA‐detecting DNA oligonucleotides (Figure 5 A), we split each oligonucleotide into a 20‐mer region that hybridises to the RNA target and a 10‐mer 5′‐tail for PCR primer binding. The two 20‐mer regions were designed to minimise self‐complementarity, which would otherwise lead to template‐independent ligation, and to be highly specific to the target as determined by BLAST^37^ alignment against a reference database. The 10‐mer sequences were designed to be non‐complementary to the target, and provide a relatively unstructured tail on which PCR primers can efficiently bind to for amplification, the latter being determined by mfold.^38^

As a proof of concept, we initially designed DNA oligonucleotides that target a 100‐mer RNA (Figure 5 A and Supporting Information, Table S9). The 3′‐amino oligonucleotide was pre‐activated with squarate ester and kept as a stock solution, while the 5′‐amino DNA oligonucleotide was left un‐activated. The two oligonucleotides were then mixed with the RNA template (3 μm to 300 pm, five‐orders of magnitude) and the ligation allowed to proceed for 15 min before analysis of the product by qPCR. Promisingly, specific RNA‐target amplification was observed, with non‐specific primer‐only amplification limiting the sensitivity of the system (Figure 5 B).

Mixing the scrambled control RNA with the specific target RNA had no impact on target RNA detection (Supporting Information, Figure S10 A). With these results in hand, we sought to push the limits of the system. To reduce primer‐only amplification, heat‐activated dNTPs were used due to the lack of a commercial heat‐activated Vent (exo‐) polymerase (Supporting Information, Figure S10 B). Furthermore, a more complex system was investigated composed of a 2664‐mer target RNA prepared through in vitro transcription that was spiked into total RNA extracted from MCF‐7 cells. The target RNA was titrated into the total RNA, enabling the determination of the dynamic range and the detection limits of the system (0.3 μm to 30 pm, five‐orders of magnitude, Figure 5 C). Pleasingly, the qPCR efficiency (110 %, R^2^=0.995) was within the generally accepted criteria of 95–110 % and gave comparable results to RT‐qPCR of the same samples (Figure 5 C). RNA detection was ultimately limited by untemplated squaramide ligation (namely, ligation in the absence of the RNA target, see Figure S10 B in the Supporting Information for negligible primer‐only amplification). This could potentially be addressed by decreasing the amino oligonucleotide concentration in the ligation reaction; however, sufficient oligonucleotide must be present to ensure the equilibrium favours hybridisation to low concentration RNA targets. Chemical modifications that enhance RNA target affinity (for example, LNA) could offer the solution to this problem.

## Conclusion

Our squaramide and urea ligation platforms expand the scope of polymerase‐compatible nucleic acid assembly and crucially provide greater flexibility in this process than previously reported methods. Through the introduction of commercially available 5′‐ and 3′‐amino groups, one‐pot ligation can generate two different linkages: 1) a urea linkage that multiple polymerases can read‐through with excellent speed, or 2) a squaramide linkage whose formation can be reversed and offers accurate read‐through under selective conditions. For the latter, the linkage can also be formed from stable pre‐activated intermediates in mild buffered conditions in the absence of small‐molecule reagents. The diversity of this platform lends itself to many applications including DNA‐encoded libraries,^2^ aptamer proximity‐ligation^39^ and DNA nano‐construct assembly.^40^ Here we have demonstrated its use in RNA detection, where it performs comparably to RT‐qPCR while offering advantages, such as removing RNA polymerase bias,^21^, ^22^, ^23^ faster speed of execution, and greater inherent target specificity.

## Conflict of interest

The authors declare no conflict of interest.

## Supporting information

As a service to our authors and readers, this journal provides supporting information supplied by the authors. Such materials are peer reviewed and may be re‐organized for online delivery, but are not copy‐edited or typeset. Technical support issues arising from supporting information (other than missing files) should be addressed to the authors.

Supplementary
