# Supplementary material for: Squaramides and Ureas: A Flexible Approach to Polymerase‐Compatible Nucleic Acid Assembly
Source: Angew Chem Int Ed Engl. 2020 May 7;59(28):11416–22. doi: 10.1002/anie.202000209 (PMC7383975; doi:10.1002/anie.202000209)
Supplement: Supplementary file 1 — Supplementary [file ANIE-59-11416-s001.pdf]

Angewandte Chemie
Eine Zeitschrift der Gesellschaft Deutscher Chemiker

Supporting Information

# Squaramides and Ureas: A Flexible Approach to Polymerase-Compatible Nucleic Acid Assembly

*Arun Shivalingam[+], Lapatrada Taemaitree[+], Afaf H. El-Sagheer, and Tom Brown**

anie_202000209_sm_miscellaneous_information.pdf

## Table of Contents

**Supplementary Methods……………………………………………………………………….S3–S10**
**Supplementary Figure 1…………………………………………………………………………S11**
**Supplementary Figure 2…………………………………………………………………………S11**
**Supplementary Figure 3…………………………………………………………………………S12**
**Supplementary Figure 4…………………………………………………………………………S12**
**Supplementary Figure 5…………………………………………………………………………S13**
**Supplementary Figure 6…………………………………………………………………………S14**
**Supplementary Figure 7…………………………………………………………………………S14**
**Supplementary Figure 8…………………………………………………………………………S15**
**Supplementary Figure 9…………………………………………………………………………S16**
**Supplementary Figure 10………………………………………………………………………S16**
**Supplementary Table 1……………………………………………………………………………S17**
**Supplementary Table 2……………………………………………………………………………S18**
**Supplementary Table 3……………………………………………………………………………S19**
**Supplementary Table 4……………………………………………………………………………S20**
**Supplementary Table 5……………………………………………………………………………S21**
**Supplementary Table 6……………………………………………………………………………S21**
**Supplementary Table 7……………………………………………………………………………S21**
**Supplementary Table 8……………………………………………………………………………S22**
**Supplementary Table 9……………………………………………………………………….S23–S24**
**Supplementary Table 10…………………………………………………………………………S24**
**Supplementary References………………………………………………………………………S25**

## Supplementary Methods

General Oligonucleotide Synthesis

*I. DNA*

DNA synthesis was performed on an Applied Biosystems 394 automated DNA/RNA synthesizer using a standard phosphoramidite cycle of detritylation, coupling, capping and oxidation using TCA (3% in dichloromethane), 1*H*-tetrazole (0.45 M in acetonitrile), Cap A (10% acetic anhydride, 10% lutidine and 80% tetrahydrofuran) / Cap B (16% N-methylimidazole in tetrahydrofuran) and iodine (0.02 M in tetrahydrofuran, pyridine and water) on a 1.0 μmole scale. Pre-packed nucleoside SynBase™ CPG 1000/110 (Link Technologies) resins or 3'-dabcyl CPG (Cambio, cat. no. 20-5912-01) packed into a twist column (Glen research) were used for synthesis β-cyanoethyl protected phosphoramidites (dA-bz, dG-ib, dC-bz and dT where bz = benzoyl and ib = *iso*-butyryl, Sigma-Aldrich) were dissolved in anhydrous acetonitrile (0.1 M) immediately prior to use. The coupling time for dA, dC, dG and dT monomers was 45 s, and 10 min for modified monomers (5'-Fluorescein, Link Technologies, cat. no. 2134). Stepwise coupling efficiencies were determined by automated trityl cation conductivity monitoring and were >98% in all cases.

*II. RNA*

RNA synthesis was performed on an Applied Biosystems 394 automated DNA/RNA synthesizer using a standard phosphoramidite cycle of detritylation, coupling, capping and oxidation on a 1.0 μmole scale. 3'-amino-modifier C7 CPG 1000 Å (Link Technologies) were packed into a twist column (Glen research) for synthesis. 2'-*O*-TC RNA phosphoramidites (A-bz, G-ib, C-ac and U where bz = benzoyl, ib = *iso*-butyryl and ac = acetyl, Sigma-Aldrich), monomers were dissolved in anhydrous toluene:acetonitrile (1:1 v/v, 0.1 M) immediately prior to use. Coupling, capping and oxidation reagents were identical to those used in DNA synthesis except a solution of 5-(ethylthio)-1*H*-tetrazole (0.25 M in acetonitrile, Link Technologies) was used as a coupling reagent. The coupling time for all monomers during TC RNA synthesis was 5 min. Stepwise coupling efficiencies were determined by automated trityl cation conductivity monitoring and in all cases were >97%.

General Oligonucleotide Deprotection

*I. DNA*

Unmodified oligonucleotides were cleaved from solid support and deprotected by exposure to a concentrated solution of aqueous ammonia in a sealed vial for 5 h at 55 °C. After drying *in vacuo*, oligonucleotides were dissolved in water and subject to further purification.

*II. RNA*

The solid support was exposed to dry ethylenediamine:toluene (1:1 v/v) for 6 h at room temperature, washed with toluene (3 x 1 mL) and dried using argon. The cleaved and deprotected RNA was eluted with water and purified further.

General Oligonucleotide Purification

*I. Desalting*

NAP™-10 columns (G.E. Healthcare Life Sciences, cat. no. GE17-0854-02) were used according to the manufacturer's instructions.

*II. RP-HPLC*

Oligonucleotides were purified using a Gilson HPLC system with ACE® C8 column (10 mm x 250 mm, pore size 100 Å, particle size 10 μm) with a gradient of buffer A (0.1 M TEAB, pH 7.5, where TEAB = triethylammonium bicarbonate) to buffer B (0.1 M TEAB, pH 7.5 containing 50% v/v acetonitrile) and flow rate of 4 mL/min.

*III. Denaturing PAGE*

Oligonucleotides was mixed with formamide (50% v/v) and loaded onto a denaturing 8 or 10% polyacrylamide gel (1x TBE buffer containing 7 M urea, W x D x H = 18 x 0.2 x 24.4 cm) and separated at 20 W for 2 h. Oligonucleotide bands were visualised under UV, excised, crushed and soaked in water (for DNA, ~15 mL) or buffer (for RNA, 50 mM Tris-HCl pH 7.5 containing 25 mM NaCl, ~15 mL) overnight at 37 °C with 900 rpm shaking. After gravity filtration to remove the gel, the oligonucleotide solutions were concentrated *in vacuo* and desalted using two consecutive NAP™-25 columns (G.E. Healthcare Life Sciences, cat. no. GE17-0852-02).

Terminal Amino Modified Oligonucleotide Synthesis

DNA synthesis was carried out on an Applied Biosystems 394 automated DNA/RNA synthesizer using identical reagents and conditions to unmodified DNA synthesis with the below exceptions:

For terminal 3'-amino oligonucleotides, pre-packed 3'-amino-dT CPG[1] resin (Cambio, cat. no. 20-2981-01) was used and coupled to standard phosphoramidite monomers.

For terminal 5'-amino oligonucleotides, the last step of oligonucleotide synthesis involved coupling of a 5'-amino-dT phosphoramidite[2] (Cambio, cat. no. 10-1932-90) for 10 min with no subsequent capping.

The structure of the resin and phosphoramidite are shown below:

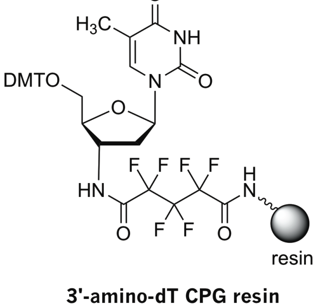


**3'-amino-dT CPG resin**

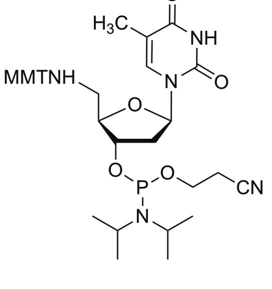

**5'-amino-dT phosphoramidite**

In both cases, stepwise coupling efficiencies were determined by automated trityl cation conductivity monitoring and were >98% in all cases.

Amino-modified oligonucleotide deprotection and cleavage from the resin was slightly altered to improve yields and purity as suggested by the supplier:

For terminal 3'-amino oligonucleotides, the resin was incubated with a concentrated solution of aqueous ammonia in a sealed vial for 18 h at 55 °C. After drying *in vacuo*, oligonucleotides were dissolved in water and RP-HPLC purified as described in the *General Oligonucleotide Purification* section.

For terminal 5'-amino oligonucleotides, prior to cleavage from the resin, the solid support was treated with diethylamine (20% in anhydrous acetonitrile) for 10 min at room temperature to suppress the formation of cyanoethyl adducts. The resin was then washed with acetonitrile (3 x 1 mL) and dried with argon before exposure to a solution (100 µL) of water in methanol (4:1 v/v) containing sodium hydroxide (0.4 M final concentration) for 3 h at 55 °C. This was to reduce acetylation of the amino groups during deprotection and cleavage of the oligonucleotide from the solid support. After cooling to room temperature, the solution was diluted with 9 volumes of water and desalted and RP-HPLC purified as described in the *General Oligonucleotide Purification* section.

<u>Oligonucleotide Mass Spectrometry</u>

All oligonucleotides were characterised by negative-mode electrospray using a UPLC-MS Waters XEVO G2-QTOF mass spectrometer and an Acquity UPLC system with a BEH C18 1.7 µm column (Waters). A gradient of methanol in triethylamine (TEA) and hexafluoroisopropanol (HFIP) was used (buffer A, 8.6 mM TEA, 200 mM HFIP in 5% methanol/water (v/v); buffer B, 20% v/v buffer A in methanol). Buffer B was increased from 0–70% over 7.5 min or 15–30% over 12.5 min for normal oligonucleotides and 50–100% over 7.5 min for hydrophobic oligonucleotides. The flow rate was set to 0.2 mL/min. Raw data were processed and deconvoluted using the deconvolution software MassLynx v4.1.

<u>Urea Ligation</u>

An exemplar ligation consists of 5'-amino-modified oligonucleotide (200 pmol, 20 µL), 3'-amino-modified oligonucleotide (250 pmol, 20 µL) and splint (250 pmol, 20 µL), sodium borate buffer (0.5 M, pH 8.5, 20 µL) and water (20 µL). This mixture was added to solid 1,1'-carbonyldiimidazole (33 mg), vortexed immediately and centrifuged before incubation for 10 min at room temperature. Note that effervescence is observed upon addition of the solution to the 1,1'-carbonyldiimidazole. Samples were then diluted with water and desalted using a NAP-10 column (G.E. Healthcare Life Sciences, cat. no. GE17-0854-02) before further purification or analysis.

Squaramide Ligation

*I. One-pot Ligation*

An exemplar ligation consists of 5'-amino-modified oligonucleotide (200 pmol, 20 µL), 3'-amino-modified oligonucleotide (250 pmol, 20 µL), splint (250 pmol, 20 µL), sodium borate buffer (0.5 M, pH 8.5, 3 µL), sodium chloride (2 M, 10 µL) and water (22 µL). 3,4-Dimethoxy-3-cyclobutene-1,2-dione (100 mM in water made fresh, 5 µL) was then added before incubation for 2 h at room temperature. Samples were then diluted with water and desalted using a NAP-10 column (G.E. Healthcare Life Sciences, cat. no. GE17-0854-02) before further purification or analysis.

*II. Amino-modified Oligonucleotide Pre-activation*

3,4-Dimethoxy-3-cyclobutene-1,2-dione (100 mM in water made fresh, 1 µL) was added to the amino-modified DNA oligonucleotide (2000 pmol, 17.8 µL) in sodium borate buffer (0.5 M, pH 8.5, 1.2 µL). After incubation for 2 h at room temperature, samples were then diluted with water and desalted using a NAP-10 column (G.E. Healthcare Life Sciences, GE17-0854-02) to yield the mono-squaramide before further use.

For RNA, 3,4-dimethoxy-3-cyclobutene-1,2-dione (100 mM in water made fresh, 30 µL) was added to the amino-modified RNA oligonucleotide (200 pmol, 13.5 µL) in sodium borate buffer (2 M, pH 8.5, 15 µL) and sodium chloride (4 M, 1.5 µL). After incubation for 2 h at room temperature, samples were then diluted with water and desalted using a NAP-10 column (G.E. Healthcare Life Sciences, cat. no. GE17-0854-02) to yield the mono-squaramide before further use.

*III. Pre-activated Oligonucleotide Quenching*

Mono-squaramide-modified DNA oligonucleotides (250 pmol, 38 µL) in water was mixed with either ethanolamine (1 M, pH 8.5, 2 µL) or Tris (1.5 M, pH 8.8, 2.5 µL) buffer. Samples were then directly analysed by UPLC-MS at the specified times.

*IV. Pre-activated Oligonucleotide Ligation*

Mono-squaramide-modified oligonucleotide (200 pmol, 20 µL), amino-modified oligonucleotide (250 pmol, 20 µL), splint (250 pmol, 20 µL), sodium borate buffer (0.5 M, pH 8.5, 3 µL), sodium chloride (2 M, 10 µL) and water (27 µL) were mixed together and left for 10 min at room temperature. Samples were then diluted with water and desalted using a NAP-10 column (G.E. Healthcare Life Sciences, cat. no. GE17-0854-02) before further use.

*V. Squaramide Oligonucleotide Cleavage*

Squaramide containing oligonucleotide (100 pmol, 25 µL) in water was mixed with an equal volume of 50% aqueous ethylenediamine (25 µL). Samples were then heated for the 3 h at 55 °C. Samples were then desalted using a NAP-10 column (G.E. Healthcare Life Sciences, cat. no. GE17-0854-02) before further use. Any variations to these conditions are described in figure captions.

qPCR Kinetics

qPCR reactions were performed using hot-start Taq (NEB, cat. no. M0495S), hot-start flex Phusion (NEB, cat. no. M0535S) or Vent (exo–, NEB, cat. no. M0257S) DNA polymerases and the primers listed in Supplementary Table 6 on a Bio-Rad CFX96. Master mixes composed of either Phusion HF buffer (5x, 4 µL), EvaGreen (20x, 1 µL, Biotium, cat. no. 31000), dNTPs (10 mM, 0.4 µL), Phusion polymerase (2 U/µL, 0.25 µL) and water (11.35 µL); or standard Taq buffer (10x, 2 µL), EvaGreen (20x, 1 µL), dNTPs (10 mM, 0.4 µL), hot-start Taq polymerase (5 U/µL, 0.1 µL) and water (13.5 µL); or ThermoPol buffer (10x, 2 µL), EvaGreen (20x, 1 µL), dNTPs (10 mM, 0.4 µL), Vent exo– polymerase (2 U/µL, 0.25 µL) and water (13.35 µL) were prepared. Note that for Taq polymerase, custom buffers as specified in figure captions were also used. This mix (17 µL) was then added to a solution of forward primer (10 µM, 1 µL), reverse primer (10 µM, 1 µL) and template (18.7 pM, 1 µL). Samples for all qPCR extension times were prepared together and stored at 4 °C before immediate sequential use. PCR thermal cycling conditions consisted of thermal activation (120 s, 95 °C), and 31 cycles of denaturation (15 s, 95 °C) and annealing/extension (30, 60, 120, 180, 240, 360 or 480 s, 60 °C), with emission recorded at the end of each extension step. Samples were excited at 450–490 nm and emission monitored at 510–530 nm. For melt curves analysis, samples were heated from 60 to 90 °C post-PCR with emission recorded every 0.5 °C using a ramp rate of 6 °C/min. Single products were confirmed by single peaks in $\partial F/\partial T$ *vs* T plots. Amplification curves were baseline corrected using the CFX96 internal analysis software.

Linear Copying Assays

Primer extension reactions were performed using Vent (exo–, NEB, cat. no. M0257S) DNA polymerase or Klenow large fragment DNA polymerase I (NEB, cat. no. M0210S) using primers listed in Supplementary Table 7 on a Bio-Rad T100. For Vent exo–, a master mix composed of ThermoPol buffer (10x, 1 µL), dNTPs (10 mM, 0.2 µL), Vent exo– polymerase (2 U/µL, 0.25 µL) and water (3.55 µL) was prepared. For Klenow, a master mix composed of NEB buffer 2 (10x, 1 µL), dNTPs (10 mM, 0.2 µL), polymerase (5 U/µL, 0.2 µL) and water (3.6 µL) was prepared. This mix (5 µL) was then added to a solution of reverse primer (10 µM, 1.5 µL), template (10 µM, 2.3 µL) and water (1.2 µL). Practically, oligonucleotide solutions and the master mix were kept on ice before mixing on ice and immediately incubating on a pre-warmed thermocycler at 60 °C (Vent exo–) or 37 °C (Klenow) for the specified times. Reactions were quenched using 20 µL of stop solution (1:3 mix of 1 mM EDTA, pH 8.0 and formamide) before storage at -20 °C. Once all samples were collected, the reaction mixture products were resolved under denaturing PAGE conditions analogous to those described in "*Oligonucleotide Purification*" section. Gels were imaged using a Syngene G:Box imager.

For mass spectrometry, the reaction was scaled up 4-fold and stopped after 1 h at 60 °C. After cooling to room temperature, phenol-chloroform (40 µL, ThermoFisher Scientific, cat. no. 15593031) was added, vortexed for 30 s and centrifuged (1,000 x g, 5 min). The aqueous layer was then transferred to a new tube, mixed with sodium acetate (3 M, pH 5.2, 4 µL,) and ethanol (132 µL), before incubating at -80 °C overnight. The samples were then centrifuged (14,000 x g, 20 min), the supernatant removed and the

pellet re-dissolved in water (10 µL). The samples were then analysed as described in the "*Oligonucleotide Mass Spectrometry*" section.

Sanger Sequencing

Squaramide or urea-containing templates (Supplementary Table 4) were PCR amplified as described in the *qPCR Kinetics* section without EvaGreen in the reaction mixture and with tailed primers that contain BbsI restriction sites (Supplementary Table 8). Samples were then purified using a QIAquick PCR Purification kit (QIAGEN, cat. no. 28104). The purified PCR product (20 ng/uL, 5 µL) was added to the mixture of pSpCas9(BB)-2A-Puro (PX459) V2.0 plasmid (Addgene, plasmid number #62988 gifted from Dr. Feng Zhang,[3] 100 ng/µL, 10 µL), BbsI-HF (20 U/ µL, 0.5 µL, NEB, cat. no. R3539S), T4 DNA ligase (400 U/ µL, 1 uL, NEB, M0202S), CutSmart Buffer (10x, 1 µL, NEB), ATP (10 mM, 2 µL, NEB) and water (0.5 µL). After incubation for 1 h at 25 °C, 2 µL of the reaction was added to NEB 5-alpha Competent *E. coli* (50 µL, High Efficiency, NEB, C2987I). The cell mixture was placed on ice for 25 min prior to heat shock for exactly 30 s at 42 °C. The cell mixture was then placed on ice for 5 min before the addition of room temperature SOC media (950 µL). After incubation for 2 h at 37 °C with 250 rpm shaking, cells were diluted 10-fold in SOC media and 50 µL was spread onto an LB agar selection plate supplemented with ampicillin (100 µg/mL). Following incubation for 16 h at 37 °C, colonies were picked and grown in a small-scale culture (5 mL) containing LB media and 100 µg/mL ampicillin for 16 h at 37 °C with 250 rpm. The plasmid DNA was extracted from the bacterial cells using the QIAprep Spin Miniprep kit according to the manufacturer's protocol (QIAGEN, cat. no. 27104). The plasmid was eluted with 50 µL water and the concentration was measured using a NanoDrop™ 2000/2000c Spectrophotometer (Thermo Fisher Scientific).

RNA Detection

*I. In Vitro Transcription of RNA Targets*

For the 100-mer RNA, a partially double-stranded DNA template was prepared by mixing the appropriate single-stranded DNA template (250 µM, 1.2 µL), the oligonucleotide complementary sequence to the T7 promoter (250 µM, 1 µL), hybridisation buffer (10x composed of 100 mM Tris-HCL pH 8.5, 500 mM NaCl and 10 mM EDTA, 4 µL) and water (33.8 µL).

For the 2,664-mer RNA, a T7 promoter-containing plasmid pcDNA3.1-hAsCpf1 (Addgene, plasmid number #69982, kindly gifted by Dr. Feng Zhang) was used as the DNA template. Prior to IVT, the plasmid (1 µg/µL, 5 µL) was linearised using PmlI restriction enzyme (2 U/µL, 2.5 µL, NEB, cat. no. R0532S) in CutSmart buffer (10x, 5 µL, NEB) and water (37.5 µL) for 1 h at 37 °C. The enzyme was then inactivated by heating the sample (20 min, 65 °C).

The RNA was prepared using the MEGAScript™ T7 *in vitro* transcription kit (Thermo Fisher Scientific, cat. no. AM1334). One reaction contained ATP (75 mM, 2 µL), CTP (75 mM, 2 µL), GTP (75 mM, 2 µL), UTP (75 mM, 2 µL), T7 enzyme mix (2 µL), reaction buffer (10x, 2 µL), dsDNA template (7.5 µM, 0.8 µL for the synthetic template or 5 µL for the linearised plasmid) and water (up to total volume 20 µL). After incubation for 16 h at 37 °C, the transcription reaction was treated with Turbo DNase (2 U/µL, 1 µL)

for 1 h at 37 °C. Next, the transcribed RNA was purified using the MEGAClear™ transcription Clean-up kit (Thermo Fisher Scientific, cat. no. AM1908). The transcription reaction was mixed with Elution Solution (80 µL), Binding Solution (350 µL) and ethanol (250 µL) and subsequently transferred to the filter cartridge. Following centrifugation (14,000 x g, 1 min), the flow-through was discarded and the sample washed using Wash Solution (2 x 500 µL). Next, Elution Solution (50 µL) was added to the filter cartridge and heated for 10 min at 70 °C. After centrifugation (14,000 x g, 1 min), the eluted RNA was collected and characterised by mass spectrometry for the 100-mer or on a 1% agarose gel stained with 0.5x SYBR gold (1x TBE running buffer, 126 V) for the 2,664-mer.

*II. Total RNA Preparation*

Total RNA was extracted from MCF-7 cells (cultured in DMEM supplemented with 10% v/v FBS) using the PureLink® RNA Mini kit (Thermo Fisher Scientific, cat. no. 12183018A) according to manufacturer's instructions. After cell dissociation using enzyme-free Hank's Balanced salt solution (Thermo Fisher Scientific, cat. no. 13150016), 1–5 x $10^6$ cells were transferred to 50 mL falcon tube and centrifuged (2,000 x g, 5 min, 4 °C). The media was discarded and the cell pellet was washed with PBS buffer. After centrifugation (2,000 x g, 5 min, 4 °C), the PBS buffer was removed and the pellet resuspended in Lysis Buffer (600 µL, containing 1% v/v 2-mercaptoethanol) by vigorous vortexing. The dispersed and lysed cell pellet was then homogenised by passing the cell lysate through a 21-gauge needle attached to a syringe (10 times). Next, an equal volume of 70% ethanol was added to the cell homogenate. After vortexing thoroughly, the mixture (700 µL) was transferred to the Spin Cartridge, centrifuged (12,000 x g, 15 s) and the flow-through was discarded. The process was repeated until no cell mixture remained. Wash Buffer I (350 µL) was then added to the Spin Cartridge. After centrifugation, the flow-through was discarded and on-column DNase digestion was performed by applying the DNase mixture containing Turbo DNase (2 U/µL, 15 µL), DNase I reaction buffer (10x, 8 µL, NEB) and RNase-free water (57 µL) onto the Spin Cartridge membrane before incubation for 15 min at room temperature. Three more rounds of washing were performed using Wash Buffer I (1 x 350 µL) and Wash Buffer II (2 x 500 µL). After further centrifugation (12,000 x g, 1 min) to remove residual liquid, RNA was eluted by adding RNase-free water (100 µL) to the Spin Cartridge, incubating for 1 min at room temperature and centrifugation (12,000 x g, 2 min). The concentration of total RNA was measured using a NanoDrop™ 2000/2000c Spectrophotometer (Thermo Fisher Scientific).

*III. RNA Detection by Squaramide Ligation*

The RNA templates were serially diluted (10-fold dilutions; 100-mer starting concentration of 2.8 µM; 2,664-mer starting concentration of 0.28 µM) in water. The appropriate 3'-amino DNA oligonucleotide was pre-activated with squarate ester as described in '*Amino Oligonucleotide Pre-activation*'. The final reactions contained mono-squaramide 3'-pre-activated oligonucleotide (2.8 µM, 1 µL), 5'-amino-modified oligonucleotide (2.8 µM, 1 µL), RNA sample (variable concentration, 1 µL), total RNA (if applicable, 100 ng, 0.4 µL), sodium borate buffer (0.5 M, pH 8.5, 0.3 µL), sodium chloride (4 M, 0.5 µL), Murine RNase inhibitor (4 U/µL, 1 µL, NEB, cat. no. M0314S) and water

(4.6 µL). After incubation for 15 min at room temperature, ethanolamine (1 M, pH 8.5, 0.5 µL) was added to quench the reaction. Prior to qPCR, the mixture was diluted 100-fold.

### *IV. RNA Detection by Reverse Transcription*

cDNA was prepared using the High-Capacity cDNA Reverse Transcription kit (Thermo Fisher Scientific, cat. no. 4368814) according to the manufacturer's protocols. 2x reverse transcription master mix consisting of MultiScribe™ reverse transcriptase (50 U/µL, 1 µL), reverse transcription buffer (10x, 2 µL), dNTPs (100 mM, 0.8 µL), random primers (10x, 2 µL), Murine RNase inhibitor (4 U/µL, 1 µL) and nuclease-free water (3.2 µL) was prepared. The mix (10 µL) was then added to a solution of IVT RNA (variable concentration, 1 µL), total RNA (100 ng, 0.4 µL) and nuclease-free water (8.6 µL). After incubation for 10 min at 25 °C and 120 min at 37 °C the temperature was raised for 5 min to 85 °C to inactivate the enzyme. Prior to qPCR, the reverse transcription reaction was diluted by 50-fold to give a final target RNA concentration that is comparable to the SQ ligation reaction.

### *V. qPCR Amplification of RNA-dependent Ligation*

The polymerase master mix consisted of Vent (exo–) DNA polymerase (2 U/µL, 0.25 µL, NEB, cat. no. M0257S), ThermoPol buffer (10x, 2 µL), EvaGreen (20x, 1 µL, Biotium, cat. no. 31000), heat-activated CleanAmp® dNTPs (10 mM, 0.4 µL, tebu-bio, cat. no. 040N-9506-2) and water (13.35 µL). The mix was then added to a solution of forward primer (10 µM, 1 µL), reverse primer (10 µM, 1 µL) and template (variable concentration, 1 µL). PCR thermal cycling conditions are composed of thermal activation (120 s, 95 °C), and 31 cycles of denaturation (15 s, 95 °C) and annealing/extension (60 s, 60 °C), with emission recorded at the end of each extension step. Samples were excited at 450–490 nm and emission monitored at 510–530 nm. For melt curves analysis, samples were heated from 60 to 90 °C post-PCR with emission recorded every 0.5 °C using a ramp rate of 6 °C/min. Single products were confirmed by single peaks in $\partial F/\partial T$ *vs* T plots. Amplification curves were baseline corrected using the CFX96 internal analysis software and standard curves plotted in MatLab R2016b.

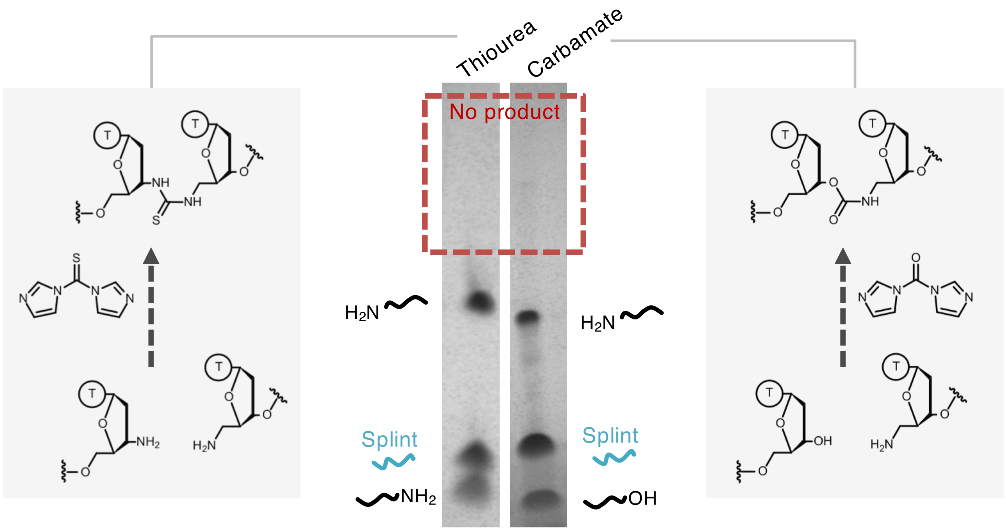


**Supplementary Figure 1 | Attempts to generate thiourea and carbamate artificial backbones by chemical ligation.** For thiourea and carbamate ligation, 20 mg of 1,1'-thiocarbonyldiimidazole or 1,1'-carbonyldiimidazole were added to a mixture of splint and reactant oligonucleotides (20 μM each) in 0.1 M NaCl. Samples were analysed after 2 h at room temperature. No ligation was observed by denaturing PAGE. The oligonucleotides used are listed in Supplementary Table 4, with the exception of unmodified 3'-OH GAGGAAGACAGCCTCGATCT, which was used in the place of Am1 for carbamate ligation.

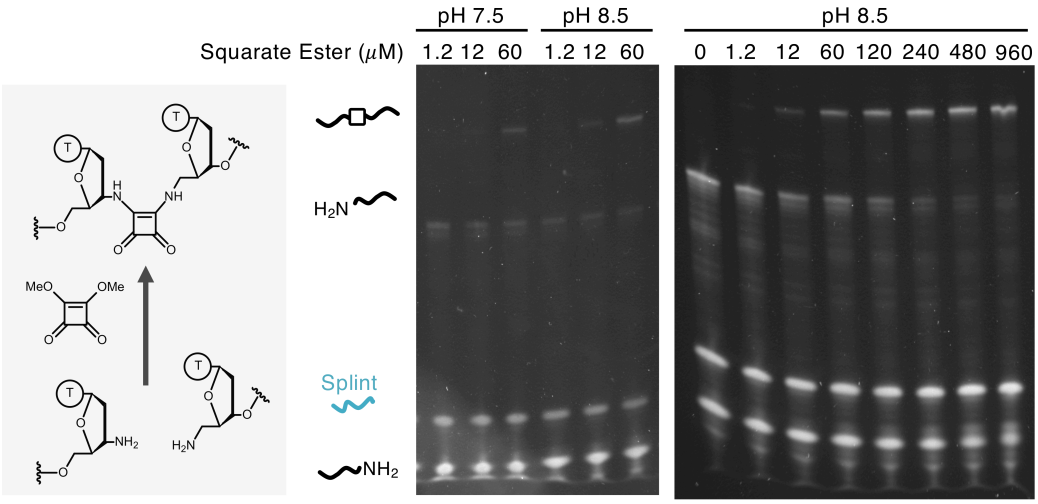


**Supplementary Figure 2 | Optimisation of one-pot squaramide ligation.** All reactions contained a mixture of splint and reactant oligonucleotides (1 μM each) in 0.1 M NaCl. For pH 8.5 reactions, 12.5 mM sodium borate buffer pH 8.5 was added. For pH 7.5 reactions, 1x PBS was added. Reactions were left for 2 h at room temperature. Note that gels were stained with SYBR gold to provide qualitative comparison of product bands. Higher pH and squarate ester equivalents gave higher amounts of ligation product. The oligonucleotides used are listed in Supplementary Table 4.

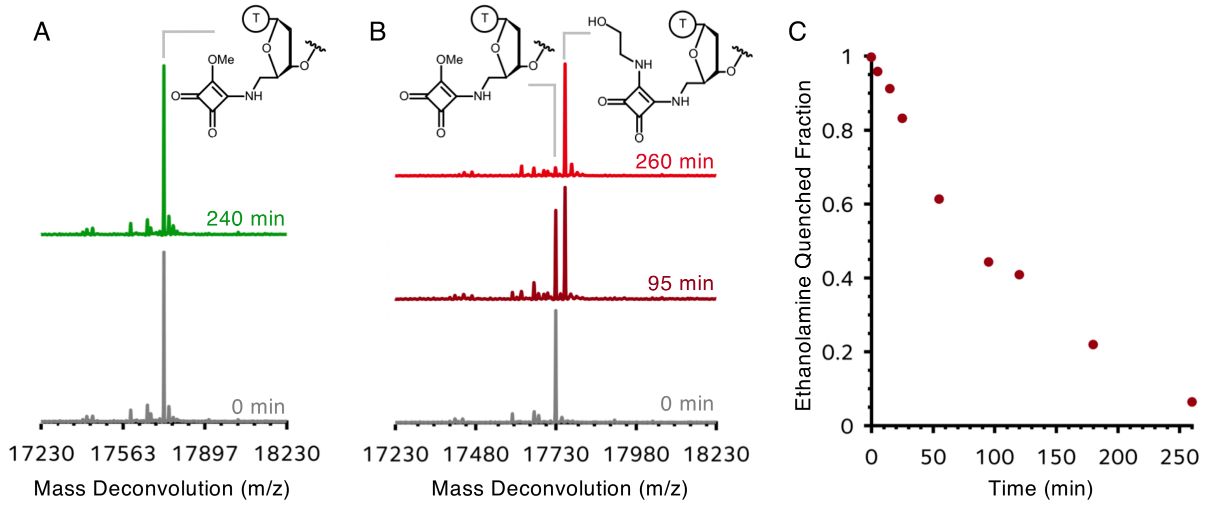


**Supplementary Figure 3 | Quenching of pre-activated mono-squaramides with small molecule amines.** (A) shows no quenching of the pre-activated mono-squaramide Am2 (M = 17729 (expected), 17730 (found)) using hindered tris(hydroxymethyl)-aminomethane (100 mM, pH 8.8), a common buffer additive after 240 min. (B) shows quenching of the mono-squaramide using primary amine ethanolamine (50 mM, pH 8.5), which reacts to form a squaramide-ethanolamine adduct (M = 17758 (expected), 17759 (found)). (C) demonstrates ethanol quenching over time (0–260 min). The ratio of the peak intensities is used to determine relative ratio of mono-squaramide to quenched products and assumes ionisation is dominated by the negative charge of the oligonucleotide. Oligonucleotide Am2 is listed in Supplementary Table 4.

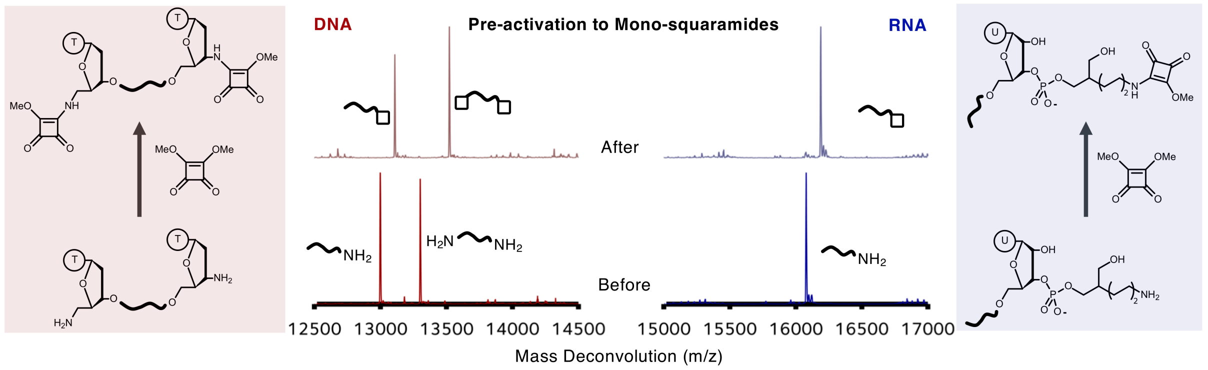


**Supplementary Figure 4 | Pre-activation of oligonucleotides (100 μM) containing terminal amino groups.** Optimised conditions described in the *'Supplementary Methods'* section were used. The DNA reaction contained a mixture of Dual-Am and Single-Am (1:1, 50 μM each), while the RNA reaction contained RNA-Am (100 μM). The reaction schematic is drawn for Dual-Am and RNA-Am. Mass spectrometry showed complete activation of the oligonucleotides with no dimerisation or cyclisation. M (Dual-Am-mono-squaramide) = 13523 (expected), 13522 (found); M (Single-Am-mono-squaramide) = 13109 (expected), 13109 (found); M (RNA-Am-mono-squaramide) = 16189 (expected), 16189 (found). The oligonucleotides used are listed in Supplementary Table 3.

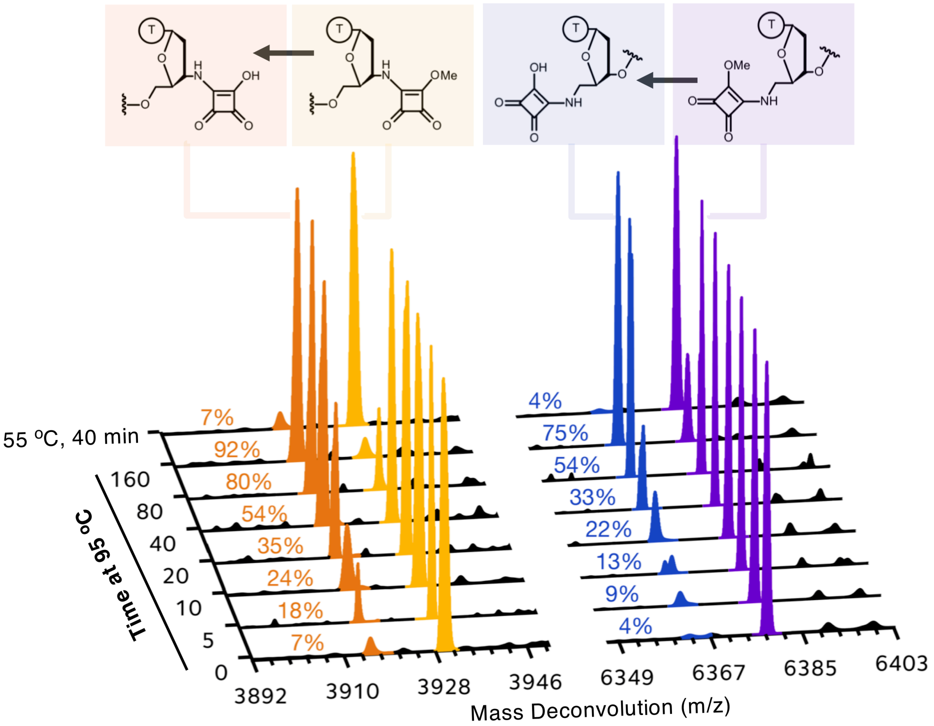


**Supplementary Figure 5 | Thermal stability of pre-activated mono-squaramide oligonucleotides.** Pre-activated F1 (M = 3929 (expected), 3930 (found)) or D2 (M = 6377 (expected), 6378 (found)) were incubated in 1x standard Taq buffer (10 mM Tris-HCl, 50 mM KCl, and 1.5 mM $MgCl_2$, pH 8.3) for the specified times and temperatures before analysis by UPLC-MS. Deconvolution of the products showed hydrolysed mono-squaramides (M (F1-hydrolysed-mono-squaramide) = 3915 (expected), 3916 (found); M (D1-hydrolysed-mono-squaramide) = 6363 (expected), 6364 (found)). Note the structures are colour-coded to the corresponding oligonucleotide peak. The ratio of the peak intensities is used to determine relative ratios of mono-squaramides to hydrolysed products and assumes ionisation is dominated by the negative charge of the oligonucleotide. The oligonucleotides used are listed in Supplementary Table 2.

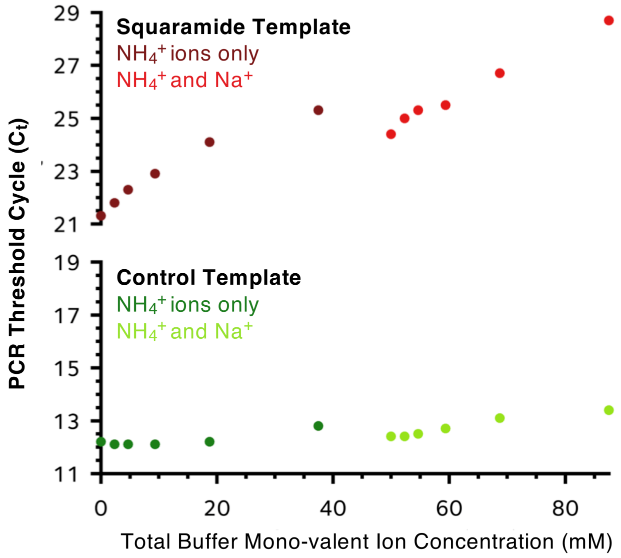


**Supplementary Figure 6 | qPCR cycle threshold ($C_t$) as a function of buffer composition for squaramide and control phosphodiester backbone-containing templates.** qPCR was performed as described in the experimental section with a buffer composed of 10 mM Tris-HCl, 1.5 mM $MgCl_2$, pH 8.3. To this buffer, $(NH_4)_2SO_4$ was supplemented (1.17, 2.34, 4.69, 9.37 or 18.75 mM). For reactions containing a mixture of $NH_4^+$ ions and $Na^+$ ions, 50 mM NaCl was added in addition to $(NH_4)_2SO_4$.

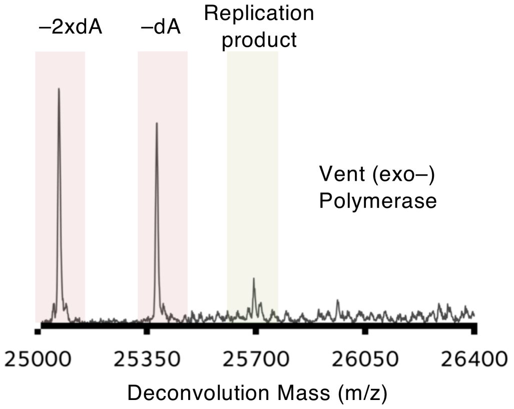


**Supplementary Figure 7 | Mass spectrometry of Klenow (exo+) polymerase replication products using the urea backbone-containing template $T_{urea}$.** Unlike Vent (exo–) polymerase, a mixture of products were obtained lacking one or two dA bases relative to the expected replication product mass (25696). This is possibly due to imperfect recognition of the local bases around the urea linkage.

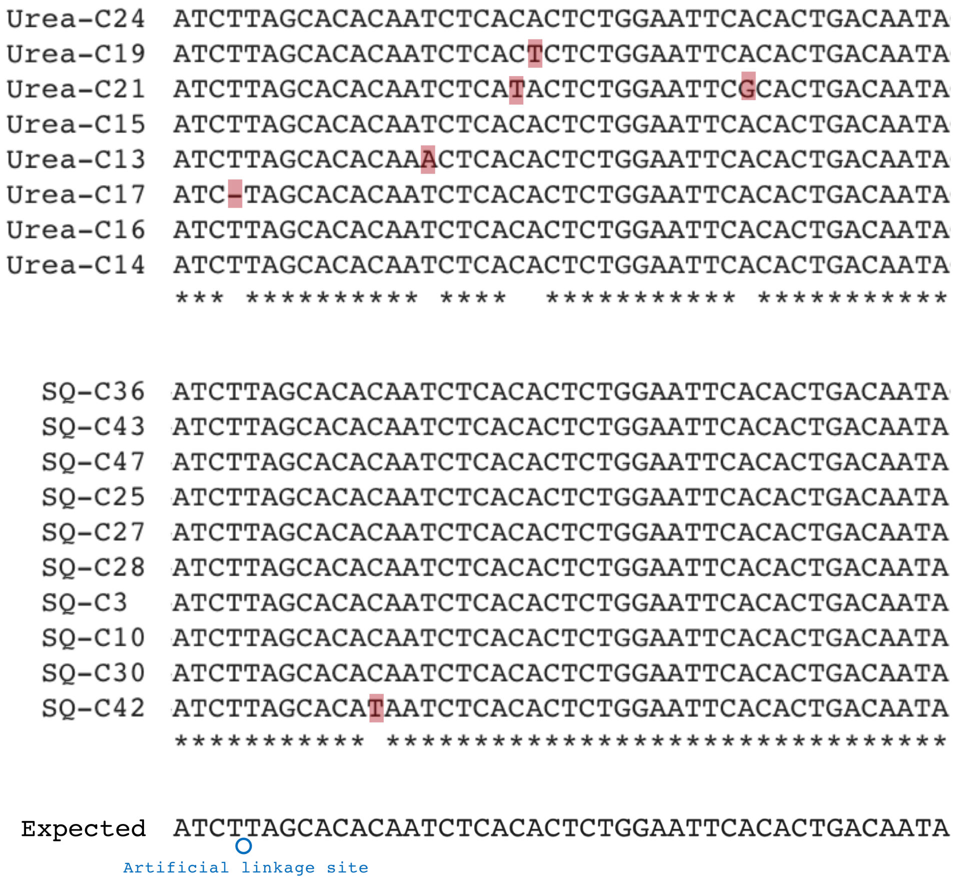


**Supplementary Figure 8 | Sanger sequencing alignments.** PCR amplicons were generated using Vent (exo–) polymerase and either squaramide (SQ) or urea backbone-containing templates. The amplicons were cloned into a vector and transformed into *E. coli*. Several colonies were randomly picked and sequenced. The expected sequence and the site of the artificial linkages are shown at the bottom. Red highlighted bases show the mutations detected. Apart from colony Urea-C17, all mismatches are non-conserved and distal to the modification site suggesting accurate read-through. The templates and primers used can be found in Supplementary Table 4 and 8 respectively.

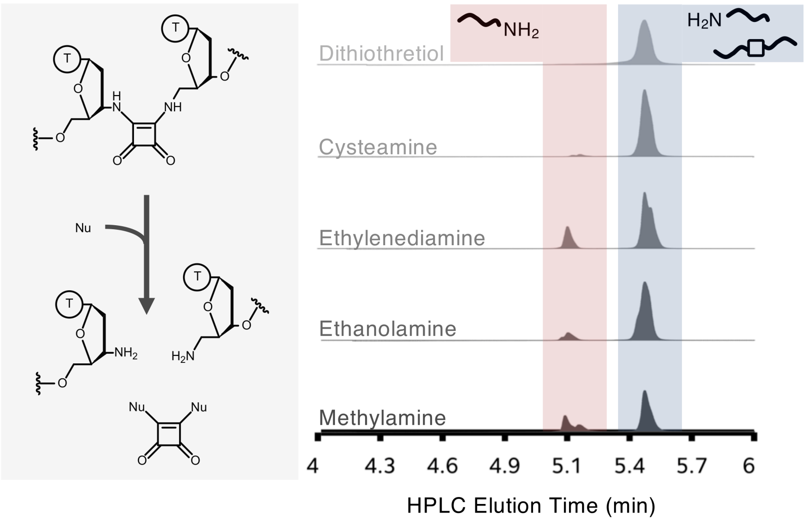


**Supplementary Figure 9 | Screen of reagents for cleaving the squaramide linkage.** 1:3 w/v of cysteamine or dithiothreitol to oligonucleotide in water, or 1:3 v/v of ethanolamine or ethylenediamine to oligonucleotide in water, or oligonucleotide in 40% aqueous solution of methylamine were heated for 1 h at 55 °C. Samples were then desalted and analysed by UPLC-MS. The blue shaded area of the UPLC trace contains uncleaved oligonucleotide and cleaved 5'-amino oligonucleotide, while the red shaded area contains the shorter cleaved 3'-amino oligonucleotide. Note that the area under the traces was normalised to 1 in order to allow qualitative comparison of the cleaved products. $T_{sq}$ from Supplementary Table 4 was used.

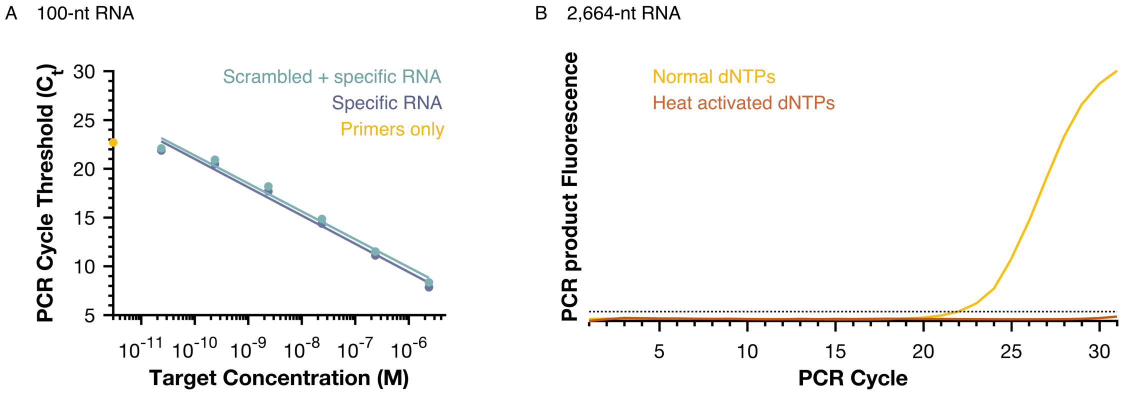


**Supplementary Figure 10 | RNA detection through templated squaramide ligation and qPCR.** (A) shows that the squaramide ligation system is still responsive to the target 100-mer RNA in the presence of an equal concentration of scrambled control 100-mer RNA. (B) illustrates qPCR amplification using only primers (i.e. no amino/mono-squaramide DNA oligonucleotides or 2,664-mer RNA target). Non-specific amplification is observed, which is significantly reduced to undetectable levels through the use of heat-activated dNTPs. The sequences used for (A) and (B) can be found in Supplementary Table 9.

**Supplementary Table 1.** Oligonucleotides used for the optimisation of ligation. The modifications and their codes are shown at the bottom of the table.

| | Code | Sequence (5'-3') | Mol. Weight (g/mol) | |
|---|---|---|---|---|
| | | | Expected | Found |
| Starting Materials | F1 | **FAM-**TAGCTCCGTC**-3'-dT-NH$_2$** | 3819 | 3819 |
| | Am3 | **5'-dT-NH$_2$-** TGACCATAGGCTCCACACCACAGAGTAAG**-C3** | 9305 | 9305 |
| | Splint 2 | TTTTTTCTTACTCTGTGGTGTGGAGCCTATGGTCAAAGACGGAGCTATTTTTT | 16346 | 16347 |
| Products | FsqC40 | **FAM-** TAGCTCCGTCT**sq**TTGACCATAGGCTCCACACCACAGAGTAAG**-C3** | 13202 | 13203 |

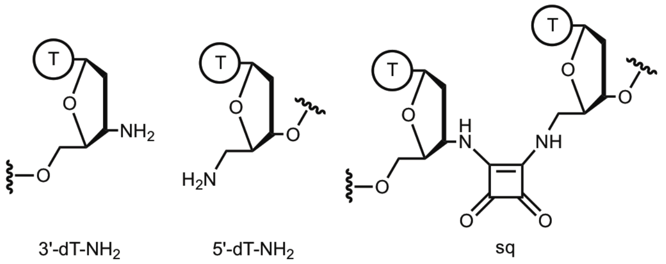


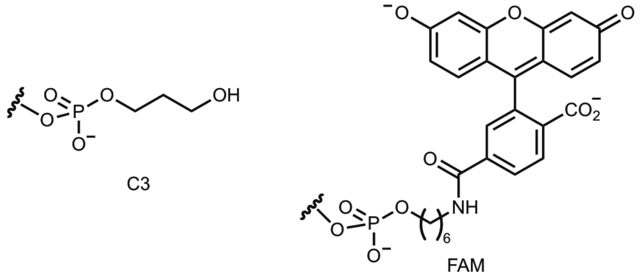

**Supplementary Table 2.** Oligonucleotides used to demonstrate reversibility of squaramide ligation. The modifications and their codes are shown at the bottom of the table.

| | Code | Sequence (5'-3') | Mol. Weight (g/mol) | |
|---|---|---|---|---|
| | | | Expected | Found |
| Starting Materials | F1 | **FAM-**TAGCTCCGTC**-3'-dT-NH$_2$** | 3819 | 3819 |
| | D2 | **5'-dT-NH$_2$-**CCAAGATCCGATGAACGA-**Dabcyl** | 6267 | 6268 |
| | Splint 3 | TTTTTTTCGTTCATCGGATCTTGGAAGACGGAGCTATTTTTT | 12912 | 12913 |
| Products | FsqD30 | **FAM-**TAGCTCCGTCT**sq**TCCAAGATCCGATGAACGA-**Dabcyl** | 10164 | 10166 |

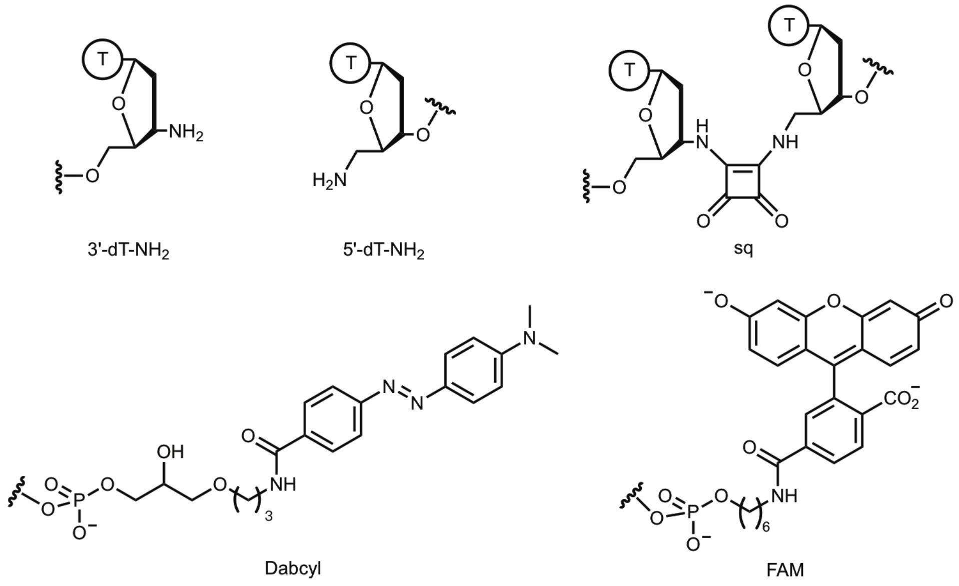

**Supplementary Table 3.** RNA and double amino-modified oligonucleotides used for pre-activation with squarate ester. The modifications and their codes are shown at the bottom of the table. Lower case bases are RNA, upper case bases DNA and underlined bases 2'-OMe modifications.

| Code | Sequence (5'-3') | Mol. Weight (g/mol) | |
|---|---|---|---|
| | | Expected | Found |
| RNA-Am | uagggguuaggguuaggguuaguuuuagagcuac gcgcaugcaacgcguu**-C7-$NH_2$** | 16079 | 16078 |
| Dual-Am | **5'-dT-$NH_2$-** CTTACTCTGTGGTGTGGAGCCTATGGTCA AAGACGGAGCTA**-3'-dT-$NH_2$** | 13303 | 13302 |
| Single-Am | CTTACTCTGTGGTGTGGAGCCTATGGTCA AAGACGGAGCTA**-3'-dT-$NH_2$** | 12999 | 12998 |

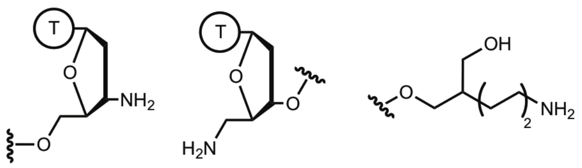


3'-dT-$NH_2$ 5'-dT-$NH_2$ C7-$NH_2$

**Supplementary Table 4.** Oligonucleotides used for the construction of squaramide and urea backbone-containing templates for linear copying and PCR assays. The modifications and their codes are shown at the bottom of the table.

| | Code | Sequence (5'-3') | Mol. Weight (g/mol) | |
|---|---|---|---|---|
| | | | Expected | Found |
| Starting Materials | Am1 | TAGAGGAAGACAGCCTCGATC**-3'-dT-NH$_2$** | 6767 | 6767 |
| | Am2 | **5'-dT-NH$_2$-**<br>AGCACACAATCTCACACTCTGGAATTCACA<br>CTGACAATACTGCAAGACACACCACTC | 17619 | 17620 |
| | Splint 1 | GAGATTGTGTGCTAAGATCGAGGCTGTC | 8699 | 8701 |
| Products | $T_{sq}$ | TAGAGGAAGACAGCCTCGATCT**sq**TAGCAC<br>ACAATCTCACACTCTGGAATTCACACTGAC<br>AATACTGCAAGACACACCACTC | 24468 | 24465 |
| | $T_{urea}$ | TAGAGGAAGACAGCCTCGATCT**urea**TAGC<br>ACACAATCTCACACTCTGGAATTCACACTG<br>ACAATACTGCAAGACACACCACTC | 24414 | 24412 |

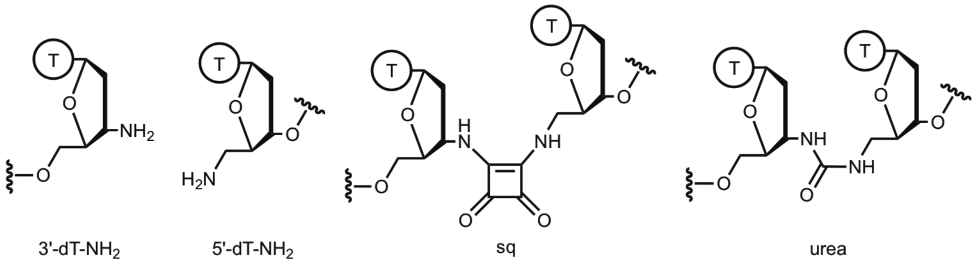

**Supplementary Table 5.** Phosphodiester (control), amide and triazole backbone-containing oligonucleotide templates. Note the amide- and triazole-modified templates were prepared as described in the reference[2] (Am 1 (TT) and Tz2 (TT)). The modifications and their codes are shown at the bottom of the table.

| Code | Sequence (5'-3') | Mol. Weight (g/mol) | |
|---|---|---|---|
| | | Expected | Found |
| $T_{tz2}$ | GCTTACGACGAAGAACGGATCT**tz2**TAGCACA CAATCTCACACTCTGGAATTCACACTGACA ATACCTCGGCCAATACACACA | 24453 | 24453 |
| $T_{amide}$ | AGCAACAGCTGGTAGACGATCT**amide**TAGCA CACAATCTCACACTCTGGAATTCACACTGACA ATACACCACAAATCCGTGCCA | 24413 | 24413 |
| $T_{control}$ | ACGTTAGCACGAAGAGGCATCTTAGCACACA ATCTCACACTCTGGAATTCACACTGACAATAC TCGCGAACACACCCAAT | 24451 | 24451 |

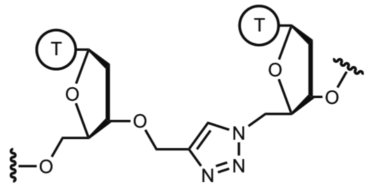

tz2

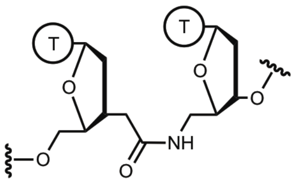

amide

**Supplementary Table 6.** PCR primers for templates listed in Supplementary Table 4 / 5.

| Template | Primer | Sequence (5'-3') |
|---|---|---|
| $T_{sq}$ / $T_{urea}$ | Forward | TAGAGGAAGACAGCCTCG |
| | Reverse | GAGTGGTGTGTCTTGCAG |
| $T_{control}$ | Forward | ACGTTAGCACGAAGAGGC |
| | Reverse | ATTGGGTGTGTTCGCGAG |

**Supplementary Table 7.** Linear copying primers used for templates listed in Supplementary Table 4 and 5.

| Template | Primer | Sequence (5'-3') |
|---|---|---|
| $T_{sq}$ / $T_{urea}$ | Reverse | FAM-TGAGTGGTGTGTCTTGCAG |
| $T_{tz2}$ | Reverse | FAM-TTGTGTGTATTGGCCGAGG |
| $T_{amide}$ | Reverse | FAM-TTGGCACGGATTTGTGGTG |
| $T_{control}$ | Reverse | FAM-TATTGGGTGTGTTCGCGAG |

**Supplementary Table 8.** PCR primers used for preparing plasmid for Sanger sequencing. Artificial backbone-containing templates can be found in Supplementary Table 4.

| Template | Primer | Sequence (5'-3') |
|---|---|---|
| $T_{sq}$ / $T_{urea}$ | Forward | TGATCAGAAGACGGCACCTAGAGGAAGACAGCCTCG |
| | Reverse | CGATGAGAAGACCTAAACGAGTGGTGTGTCTTGCAG |

**Supplementary Table 9.** Oligonucleotides used for IVT RNA detection using a squaramide ligation-based detection step. The modifications and their codes are shown at the bottom of the table. Lower case bases are RNA while upper case bases are DNA. ppp = triphosphate.

| | Code | Sequence (5'-3') | Mol. Weight (g/mol) | |
|---|---|---|---|---|
| | | | Expected | Found |
| 100-mer IVT RNA Target | T7-promoter | TCTAATACGACTCACTATAG | 6060 | 6059 |
| | IVT Template (Specific) | ATATTAGTTGACTCTGATCTCGCGTGAAGTATCGGCACAGCAACGTGATTTGACCATAGGCTCCACACCCAAATGGAGCAGAATCATGTGTGCTTCACCCTATAGTGAGTCGTATTAGA | 36704 | 36707 |
| | IVT Template (Scrambled) | ATATTAGTTGACTCTGATCTCGCGTGACCCGACAGATATAGCAGAGACCGCACTCGCGATAACTTTCTGCAAATGGAGCAGAATCATGTGTGCTTCACCCTATAGTGAGTCGTATTAGA | 36704 | 36708 |
| | IVT Specific | **ppp**gggugaagcacacaugauucugcuccauuugggguguggagccuauggucaaaucacguugcugugccgauacuucacgcgagaucagagucaacuaauau | 32384 (+Mg) | 32385 |
| | IVT Scrambled | **ppp**gggugaagcacacaugauucugcuccauuugcagaaaguuaucgcgagugcggucucugcuauaucugucgggucacgcgagaucagagucaacuaauau | 32384 (+Mg) | 32385 |
| | 100-Am3 | CGAAGTACTGAGTATCGGCACAGCAACGTGA-**3'-dT-NH$_2$** | 9881 | 9882 |
| | 100-Am3-SQ | CGAAGTACTGAGTATCGGCACAGCAACGTGA-**3'-dT-mono-sq** | 9991 | 9992 |
| | Am5 | **5'-dT-NH$_2$-**TGACCATAGGCTCCACACCACAGAGTAAG | 9305 | 9305 |
| | Primer F | CGAAGUACTGAGTATCGGC | 5853 | 5854 |
| | Primer R | CTTACTCTGTGGTGTGGAG | 5866 | 5865 |
| 2,664-mer IVT RNA Target | Cpf1-Am3 | AGCAGCTGTACTAGATCTCGGCGTGTCTC-**3'-dT-NH$_2$** | 9188 | 9189 |
| | Cpf1-Am3-SQ | AGCAGCTGTACTAGATCTCGGCGTGTCTC-**3'-dT-mono-sq** | 9298 | 9300 |
| | Cpf1-Am5 | **5'-dT-NH$_2$-**ATTGATGGCATCGGTCAGGGCTACTTAGC | 9252 | 9253 |
| | Cpf1-Primer F | AGCAGCTGTACTAGATCTCG | 6117 | 6118 |
| | Cpf1-Primer R | GCTAAGTAGCCCTGACCG | 5485 | 5485 |
| | Cpf1-RT-Primer F | GGACAGACAACCTGACCG | 5503 | 5503 |
| | Cpf1-RT-Primer R | ACAGGCCCTTGTAGATCTC | 5764 | 5765 |

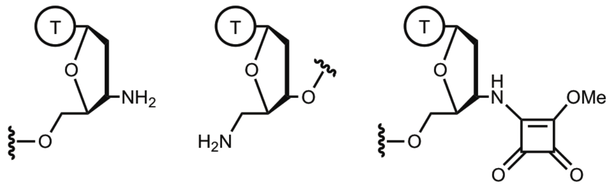


3'-dT-NH$_2$ 5'-dT-NH$_2$ 3'-dT-mono sq

**Supplementary Table 10.** Summary of squaramide ligation couplings efficiencies and calculations. The data is a summary of values presented in Figure 2B, 2D, 2E and 4B. For denaturing PAGE gels, AuC represents band intensity determined using ImageJ. For UPLC UV traces, AuC was determined by integration of area under the trace. AuC = Area under Curve. f = Fraction. * = both are a measure of ligation efficiency for the same reaction therefore the average is shown in Figure 2E.

| Technique | Figure | Oligo State | AuC | Yield (%) | Equation |
|---|---|---|---|---|---|
| Denaturing PAGE FAM-Label | Fig. 2B | Unreacted | 3689 | 71.37 | $f_{reacted}/f_{total}$ x 100 |
| | | Reacted | 9195 | | |
| Denaturing PAGE FAM-Label 3'-Activated | Fig. 2D | Unreacted | 959 | 88.54 | $f_{reacted}/f_{total}$ x 100 |
| | | Reacted | 7406 | | |
| Denaturing PAGE FAM-Label 5'-Activated | Fig. 2D | Unreacted | 1713 | 79.57 | $f_{reacted}/f_{total}$ x 100 |
| | | Reacted | 6670 | | |
| PAGE UV Shadowing | Fig. 2E (Middle Band) | Control | 10277 | 68.72* | 1 - $f_{unreacted}/f_{control}$ x 100 |
| | | Unreacted | 4677 | | |
| | Fig. 2E (Bottom band) | Control | 5992 | 74.23* | |
| | | Unreacted | 2080 | | |
| UPLC UV Trace | Fig. 4B (Red peak) | Purified | 78.31 | 100.00 | $f_{state}/f_{purified}$ x 100 |
| | | Uncleaved | 3.95 | 5.04 | |
| | | Re-ligation | 55.7 | 71.13 | |

## Supplementary References

[1] R. P. Glinski, M. S. Khan, R. L. Kalamas, M. B. Sporn, *J. Org. Chem.* **1973**, *38*, 4299–4305.
[2] A. Shivalingam, A. E. S. Tyburn, A. H. El-Sagheer, T. Brown, *J. Am. Chem. Soc.* **2017**, *139*, 1575–1583.
[3] F. A. Ran, P. D. Hsu, J. Wright, V. Agarwala, D. A. Scott, F. Zhang, *Nat. Protoc.* **2013**, *8*, 2281–2308.
